# Acute liver injury in two workers exposed to chloroform in cleanrooms: a case report

**DOI:** 10.1186/s40557-014-0049-5

**Published:** 2014-11-04

**Authors:** Young Joong Kang, Jungho Ahn, Yang-In Hwang

**Affiliations:** Occupational Safety and Health Research Institute, Korea Occupational Safety and Health Agency, 400, Jongga-ro, Jung-gu Ulsan, Republic of Korea; Gyeonggi Bukbu Area Office, Korea Occupational Safety and Health Agency, 140, Chudong-ro Uijeongbu-si, Gyeonggi-do Republic of Korea

**Keywords:** Chloroform, Trichloromethane, Liver toxicity, Toxic hepatitis, Cleanroom

## Abstract

We report 2 cases of hepatotoxicity in cleanroom workers due to high retained chloroform air concentrations. The women, aged 34 and 41 years, who had been working in a medical endoscopic device manufacturer as cleanroom workers for approximately 40–45 days suffered severe liver damage. Two measured time-weighted averages of the chloroform concentration in the air in the cleanroom were 82.74 and 64.24 ppm, which are more than 6 times the legal occupational exposure limit in Korea. Only 7% of the cleanroom air was newly introduced from outside. The clinical courses of these cases and workplace inspection, led us to conclude that both cases of hepatotoxicity were caused by chloroform exposure.

## Background

Chloroform (CHCl_3_, CAS No. 67-66-3), also called trichloromethane or methylchloride, is a volatile organic compound that is a noninflammable and colorless liquid with a distinct odor and slightly sweet taste. As chloroform is very volatile and soluble in organic solvents, it is used as a solvent and cleanser for plastic compounds as well as an acrylic adhesive. The hepatotoxicity of chloroform is well known; Meyer and Pessôa first recognized the toxicity of chloroform to humans in 1923 [1].

Cleanrooms, which are typically used in manufacturing and scientific research, are special environments with strictly controlled levels of pollutants such as dust, airborne microbes, and aerosol particles; they have specifically controlled conditions including temperature, humidity, pressure, and airflow. Cleanrooms are classified according to the number of dust particles contained in a given volume of air, which is maintained by indoor air recirculation and filtering. It is critical to remove all hazardous materials generated in cleanrooms when chemical substances are handled inside, because the accumulation of hazardous materials could be very dangerous to workers with repeated exposure [2]. Thus, cleanroom workers involved in the manufacturing of microelectronic devices are exposed to several risks [3-5].

Here, we report 2 cases of hepatotoxicity due to chloroform exposure in a cleanroom used for the manufacturing of medical endoscopic devices.

## Case presentation

### Case 1

A 34-year-old woman who had been working in a medical endoscopic device factory for approximately 40 days developed nausea, vomiting, and jaundice. Approximately 1 month after beginning working in that factory, she developed abdominal discomfort, systemic pruritus, and dark urine; her sclera changed to icteric. However, she thought these symptoms were not serious. She worked several more days without any medical treatment. She rested at home after her last duty, but her symptoms worsened. She visited the hospital and was admitted to the Department of Gastroenterology because of suspected liver damage. She was healthy before hospitalization. She had no specific medical history or familial or genetic problems. She had taken oral contraceptives for 4 months but stopped them 1 month prior. She did not take any other drugs, health supplements, or herbs. She had no history of smoking and rarely consumed alcohol.

She worked in the cleanroom of medical endoscopic device manufacturer, assembling devices with chloroform adhesives. It took about a month when she got first symptoms related to her liver damage. She worked several more days, then her symptoms worsened. She worked about 40 days before admission. To protect the goods from pollutants, she wore cleanroom garments during work but did not wear any protective equipment.

Her laboratory data on admission were as follows: white blood cell count, 4,510/mm^3^; red blood cell count, 446/mm^3^, hemoglobin, 13.1 g/dL; platelet count, 265,000/mm^3^; aspartate aminotransferase (AST), 350 IU/L; alanine aminotransferase (ALT), 499 IU/L; lactate dehydrogenase 450 IU/L; alkaline phosphatase, 66 IU/L γ-glutamyl transpeptidase, 45 IU/L; total bilirubin, 8.5 mg/dL; direct bilirubin, 6.2 mg/dL; PT, 14.3 s (PT-INR, 1.32; percent, 62.9); and aPPT, 35.6 s. Test results for hepatitis-associated antigen and antibody excluded viral and autoimmune hepatitis: anti-HAV IgG (+), anti-HAV IgM (−), HBs Ag (−), anti-HBs Ab (+), anti-HCV (−), HIV Ag/Ab (−), anti-smooth muscle Ab (−), FANA (−), AMA (−), and anti-LKM Ab (−). Abdominal ultrasonography examination was normal.

She responded to hydration and conservative management. Her symptoms subsequently lessened, and AST/ALT levels stabilized. On hospital day 4, AST/ALT declined to 179/309. By Out Patient Department followed up, AST/ALT declined to 46/83. AST/ALT values are shown in Table 1. Her condition returned to normal, but she did not return to her work place.

**Table 1:** **Liver enzymes**

**Values (Days after admission)**					
Case 1	AST/ALT	359/499 (0)	295/436(1)	179/309(3)^*^	46/83 (11)^†^
Case 2	AST/ALT	767/1420(0)	312/739(3)	140/341(7)^*^	40/98 (14)^†^

^*^Last laboratory data during the course of hospitalization.

^†^Out Patient Department follow up visit.

### Case 2

A 41-year-old woman who had been working in a medical endoscopic device factory for 45 days visited the Department of Gastroenterology complaining of myalgia, febrile sensation, nausea, vomiting, and abdominal discomfort over the previous 4 to 5 days; her symptoms worsened and urine color changed 2 days prior. She was admitted to the Department of Gastroenterology with suspected liver damage. Regarding medical history, she underwent a routine health examination 4 months ago and was diagnosed with reflux esophagitis and osteoporosis; she did not take medications for either. She took NSAIDs for 3 months for back pain but had rarely taken them in the last month. She underwent hysterectomy and appendectomy 5 and 25 years previously, respectively. She had not recently taken any drugs, herbs, or health supplements. She had no specific familial or genetic problems. She did not smoke. She reported consuming alcohol socially once per month.

She also worked in the same medical endoscopic devices manufacturer as the patient in case 1 and performed the same duty. It was 40 days after beginning working in the cleanroom when she got first symptoms related to liver damage. She worked 45 days before hospitalization. She also wore cleanroom garments during cleanroom work but did not wear protective equipment.

Her laboratory data were as follows: white blood cell count, 6,450/mm^3^; red blood cell count, 460/mm^3^; hemoglobin, 14.9 g/dL; platelet count, 186,000/mm^3^; AST, 767 IU/L; ALT, 1,420 IU/L; lactate dehydrogenase, 540 IU/L; alkaline phosphatase, 131 IU/L; total bilirubin, 3.6 mg/dL; PT, 10.4 s (PT-INR, 0.91; percent, 131.2). Hepatitis-associated antigen and antibody results were as follows: anti-HAV IgG (+); anti-HAV IgM (−); HBs Ag (−); anti-HBs Ab (+); anti-HCV (−); HIV Ag/Ab (−); anti-smooth muscle Ab (−); AMA (−); anti-LKM Ab (−); FANA (1:160) cytoplasmic pattern; ferritin, 159 ng/mL; iron, 157 μg/dL; total iron binding capacity, 200 μg/dL; serum IgG, 1,409.0 mg/dL; serum IgA, 231.0 mg/dL; and serum IgM, 127.0 mg/dL. She exhibited with mild fatty liver on abdominal ultrasonography. Although FANA titer was positive, this finding is not usually present alone in auto-immune hepatitis; It usually presents with other auto-immune antibodies and anti-smooth muscle antibody [6]. FANA also can be presented in drug-induced hepatitis and other liver diseases [7,8]. Immunoglobulin G levels in blood were not elevated [9]. Therefore, this case was diagnosed as chloroform-induced hepatitis.

She was also treated with hydration and conservative management. Both subjective symptoms and laboratory data subsequently improved. Her AST/ALT declined to 140/341, 7 days after hospitalization. On Out Patient Department follow up, Her liver enzyme values subsequently declined to 40/98. The AST/ALT values are shown in Table 1. Her condition returned to normal, but she did not return to her work place, either.

### Workplace inspection

**Figure 1:**
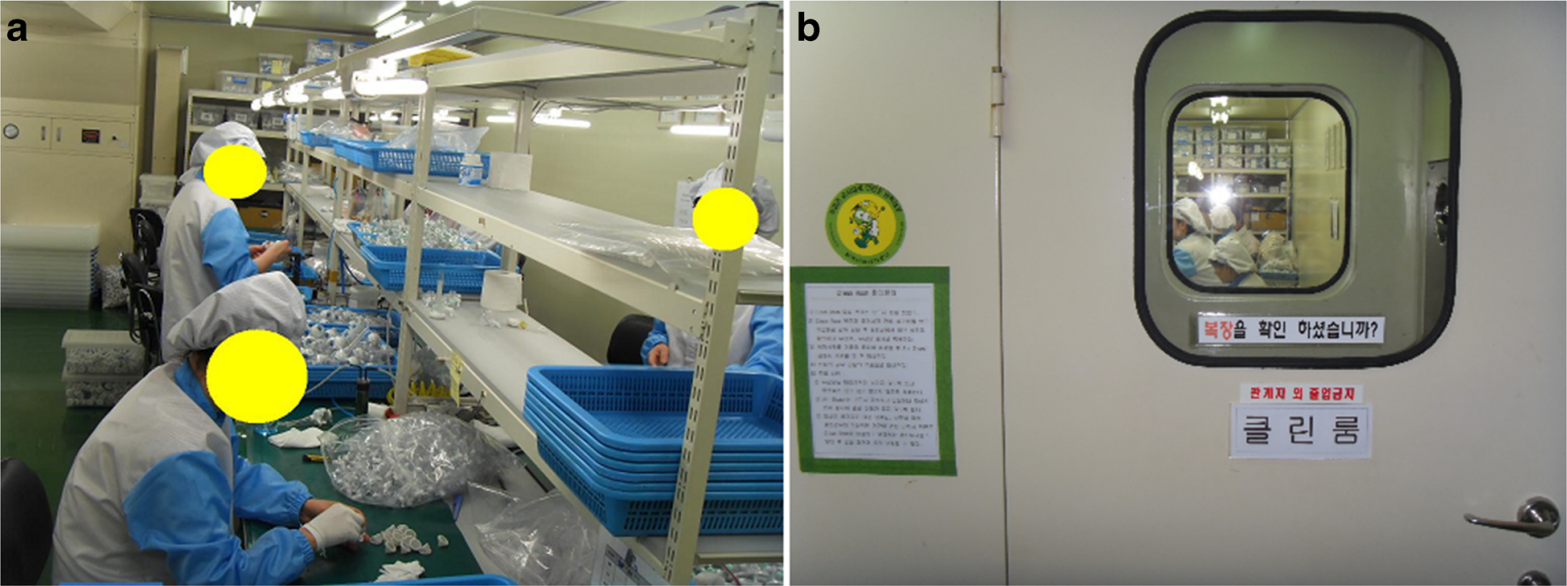
**Cleanroom workers inside the cleanroom. a**. Inside the cleanroom, workers are assembling with components of endoscopic devices with chloroform adhesives. **b**. The cleanroom is covered up tightly.

Both patients worked at the same factory that manufactured medical endoscopic devices. This small business is consists of 12 workers. 4 workers are mainly assigned to the cleanroom work (Figure 1). The cleanroom maintained particulate-free air through use of high-efficiency particulate air (HEPA) filters applying turbulent airflow principles. This cleanroom was maintained according to ISO 14646–1 class 5. There had not been any air circulation system except a HEPA filter.

During work, the cleanroom workers used 99% chloroform as an adhesive and 98% ethanol as a cleanser. They used chloroform adhesive by pouring it from large bottles into 50-mL vessels. They subsequently immersed their parts of the device in the chloroform adhesive vessel or applied chloroform to a part of the devices with a micro-feeder. The mean daily amount of chloroform used was 50 mL. There had not been any installed ventilation system. Two time-weighted average (TWA) measurements of the chloroform air concentration in the cleanroom were 82.74 and 64.24 ppm. These values were much higher than those measured in the other workshop constructed from a shipping container, where almost the same process was performed with the same chloroform adhesive. However, the container workshop had natural ventilation through windows (Table 2). TWA measurements of the chloroform in the container box worksite were 5.34 and 3.34 ppm. The Korean occupational exposure limit is 10 ppm for 8 hours TWA; meanwhile, the US legal limit, the permissible exposure limit, which is a ceiling limit that is not to be exceeded even momentarily, is 50 ppm. Even though the workers used just 50 mL chloroform daily, the air concentrations hugely exceeded regulations.

**Table 2:** **Concentrations of volatile organic compounds**

**Sampling area**	**Sampling time (min)**	**Chemical**	
		**Chloroform (ppm)**	**Ethanol (ppm)**
Cleanroom	337	82.74	160.61
	337	64.24	98.71
Container workshop	335	5.35	21.18
	338	3.34	35.34
OEL* (TWA^†^)		10	1,000

^*^ *OEL*, Occupational exposure limit.

^†^ *TWA*, Time-weighted average.

**Figure 2:**
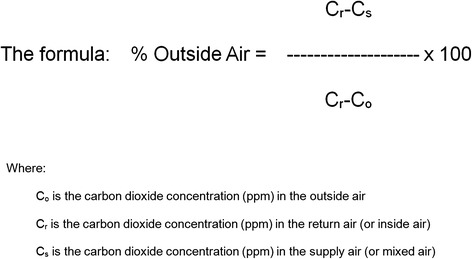
**The percentage of outdoor air recirculated can be calculated by inputting the tracer values into this formula.**

We estimated the proportion of outside air supply to determine the adequacy of ventilation in the cleanroom, because direct measurement of the amount of outside air entering is difficult and inaccurate. The percentage of outdoor air recirculated can be calculated using carbon dioxide as a tracer gas on the basis of the mass balance of air and tracer in a single area [10]. Therefore, we determined the outside air ventilation rate by inputting the tracer values into the formula below (Figure 2). The concentrations of carbon dioxide in the cleanroom, outside, and mixed air in the air supply duct were measured twice (Table 3). The values obtained were input into the following formula to determine the proportion of outside air intake in the cleanroom. The values obtained were subsequently used to calculate the percentage of outside air that diluted the air inside the cleanroom [10,11].

**Table 3:** **Carbon dioxide concentrations the cleanroom**

**Measuring point**	**CO**_**2**_ **concentration (ppm)**	
	**10:25 AM**	**4:00 PM**
Supply air	2,020	2,280
Recirculation air	2,140	2,400
Outdoor air	560	570

The proportion of air supply from outside was from 6.6–7.6%; thus, more than 90% of the inside air was recirculating and consequently accumulating hazardous chemicals. A survey of cleanroom work environments in the microelectronic industry in Korea in 2009 reports the average proportion of air supply from outside was 30% [2]. The Concentration of ethanol in the air in the cleanroom was measured, too. Two time-weighted average (TWA) measurements of the ethanol air concentration in the cleanroom were 160.61 and 98.71 ppm. It is much lower than Korean occupational exposure limit 1,000 ppm per 8 hours TWA. The factory was small with only 12 workers. All workers were probably exposed to chloroform in their work place, but only 4 workers were assigned to work in the cleanroom. No other major chemical, physical, or biological hazard except chloroform was identified in the workplace in question. No other workers except these 2 cases were known to have newly diagnosed liver disease. They knew the chloroform in their adhesive was regulated chemical but did not realize the regulation aimed to prevent human toxicity. The owners did not provide any protective equipment to workers because they thought chloroform exposure would not exceed regulations. After the diagnosis of these 2 cases and an inspection, they closed their factory and re-opened it under a new name while still manufacturing the same goods. It is assumed that they were afraid of additional inspections or regulations. They renovated their cleanroom with local exhaust ventilation. They also changed the adhesive to one that does not contain chloroform.

## Conclusion

The investigation of the conditions of the cleanroom where the 2 patients with hepatotoxicity worked revealed that there was only a HEPA filter for particulates and no adequate ventilation system. Thus, chloroform accumulated excessively in the cleanroom because of repetitive inner circulation of the particulate filtered air and the absence of fresh air inflow. These findings indicate that without an appropriate ventilation system to maintain sufficient inflow of fresh air, even small amounts of volatile compounds can accumulate in high concentrations in cleanrooms and cause severe health damage to workers.

Particulate levels in cleanrooms are usually maintained for the quality control of the goods made. However, conditions may insufficient to protect workers’ health. Outside air to dilute indoor air is an essential element for maintaining air quality in workplace but makes it hard to maintain air quality in the cleanroom. It can increase costs associated with heating, cooling or humidity and also can allow pollutants in, which increase the number of defective goods produced. Cleanroom manufactures may neglect to maintain adequate ventilation amounts of outside air to lessen the associated costs. The main purpose of cleanroom garments is to protect sensitive goods from contamination by workers and not to protect the workers themselves. Cleanrooms are usually restricted areas. Therefore, when workers enter cleanrooms, they usually spend many hours inside. Hence, without proper protective equipment and ventilation, cleanroom workers will be continuously exposed to hazardous conditions [2,4].

The majority of chloroform produced is used to make HCFC-22. It can be released into the air as a byproduct of its formation in the chlorination of drinking water, sewage, and swimming pools. It also can be produced as a byproduct of bleaching paper. Chloroform may also be emitted in a vehicle exhaust [12]. Chloroform used to be used as an extraction solvent for fats, oils, and other products; dry cleaning remover; fumigant and anesthetic [13]. The major effect of acute inhalation of chloroform is central nervous system depression. At concentrations from 1,500-30,000 ppm, chloroform exposure can induce anesthesia; at concentrations exceeding 40,000 ppm, it can be fatal. Chronic inhalation of chloroform in humans results in hepatotoxicity and central nervous symptoms such as depression and irritability. Meanwhile, chronic oral exposure to chloroform in humans results in effects on the blood, livers and kidneys [13]. Chloroform is classified as a Group 2B, probable carcinogen [14]. The hepatotoxicity of organic solvents was first recognized in the late 19^th^ century. In particular, the hepatotoxicity of chloroform was first recognized in 1923 [1,15,16]. Bomski et al. and Gambini et al. subsequently reported chloroform-induced hepatotoxicity in various industries [17,18]. Meanwhile, Phoon et al. report 2 outbreaks of chloroform-induced hepatotoxicity involving 31 workers exposed to chloroform at work; among them, 13 and 18 workers were exposed to approximately 14.4–33.3 and 19.6–50.4 ppm, respectively. Regarding duration, 5 and 28 workers were exposed for <1 and <3 months, respectively [19]. There is another case series of 13 jaundice patients exposed to chloroform (400 ppm) inhalation at their workplaces [20]. In Korea, a case of suspected chloroform-induced hepatotoxicity was reported in a laboratory engineer in 2012 even though the chloroform concentration only ranged from 3.155–9.037 ppm [21]. Although chloroform exhibits a dose-dependent effect, it also causes unpredictable idiosyncratic toxicity [22].

The mechanism of liver injury is most likely the result of changes in metabolic rate of the liver. Chemicals are generally metabolized in the liver, consequently producing toxic metabolites [12]. This will not occur unless the absorbed toxic material exceeds the detoxification capacity of the liver. Glutathione and CYP450 play significant roles in the detoxification process. A reduction in the ability of CYP450 to detoxify solvents increases the percentage of lipids in the liver [16]. Simultaneous exposure to ethanol, which reduces CYP450 activity, may result in increased chloroform retention [23]. In the present cases, 98% ethanol was used as a cleanser. The 2 TWA air concentrations of ethanol were 98.71 and 160.61 ppm, respectively (ethanol occupational exposure limit: 1,000 ppm TWA). Therefore, ethanol exposure likely exacerbated chloroform retention in these cases.

Many studies suggest exposure to organic solvents can cause hepatotoxicity [24,25]. Despite this knowledge, occupational organic solvent exposure-induced hepatotoxicity is rarely diagnosed or even suspected [26]. Several factors contribute to solvent hepatotoxicity, including species differences, hepatic blood flow, protein binding, age, nutrition, etc. [12]. Therefore, it is difficult to distinguish the precise effect of occupational hepatotoxicity from other hepatotoxicity-related factors [16]. Furthermore, it is virtually impossible to completely control chronic liver damage among workers because of nonspecific symptoms and signs as well as the low sensitivity of conventional liver enzyme tests [27]. It is occasionally possible to assume the dose-dependent toxicity, but impossible to predict the exact dose–response relationship. Furthermore, epidemiological analyses of the effects of solvents on the liver are very difficult. Therefore, it is important to focus on individual cases.

To confirm a diagnosis of chemical induced toxic hepatitis, other forms of hepatitis and other disorders with similar symptoms should be ruled out. The diagnostic criteria are as follows: occupational exposure preceding liver damage; liver enzymes at least double the upper limit of normal level; and other causes of liver disease excluded [28,29]. In the 2 cases reported herein, workplace chloroform exposure preceded the patients’ recognition of symptoms. Their liver enzyme levels increased to more than 3 times the upper limit of the normal range. Other causes of hepatitis were ruled out during the clinical courses. In conclusion, severe liver dysfunction was observed in 2 female workers who had been exposed to high chloroform concentrations in the same cleanroom, which had an inappropriate ventilation system.

The incidence of occupational organic solvent exposure-induced hepatotoxicity is not unprecedented. However, it is also very unpredictable, making epidemiological analysis very difficult. Therefore, it is still important to focus on individual cases. Chloroform, which is comparatively unknown as a hepatotoxic organic solvent, requires more attention. Above all, the environments of cleanroom worksites require more attention. The findings of the present cases indicate that without an appropriate ventilation system to maintain adequate indoor air quality, small amounts of volatile compounds can create a very dangerous work environment in cleanrooms. The chloroform concentration measured in the cleanroom in the present cases was much higher than that in the container workshop where the same chemicals were used; although it did not have any ventilation system, it had ventilation thorough windows.

## Consent

Written informed consents were obtained from the patients for publication of this Case report and any accompanying data.
